# An Insurance Incident

**Published:** 1871-04

**Authors:** 


AN INSURANCE INCIDENT.—Under this heading, Dr. John B. Cowan relates, in the last number of the *Glasgow Medical Journal*, the case of a man aged 49, who, having proposed for assurance and being unable to state whether he ever had smallpoxor been vaccinated, had himself vaccinated on the left arm. The operator states that the patient had "the original marks of vaccination as distinct as you could expect to find them in a man between forty and fifty years of age who had not been vaccinated since childhood. I vaccinated him on the left arm; three places all took thoroughly, and he went through the whole process satisfactorily." Seven months after re-vaccination, this man died of virulent smallpox. Dr. Cowan states that "in the experience of an assurauce company which has carried on business for upwards of thirty years, no similar incident has occurred." [Vaccination cannot be expected to protect more thoroughly than smallpox itself, and cases of death in a second attack of smallpox are not unheard of. We know that during the last epidemic in Edinburgh some of the worst cases were in those who had smallpox previously, and some of these died. [We have seen very sharp cases of secondary vaccinia caught from the cow within a comparatively short interval of each other. And interesting as the above case may be in a pathological point of view, it in no way invalidates the protective power of vaccination, or contradicts the ordinary view that death almost never occurs from smallpox after properly-performed vaccination—on the contrary, it rather confirms it.]—*Edenburg Medical Journal.*

